# Clinical Utility of Microarrays: Current Status, Existing Challenges and Future Outlook

**DOI:** 10.2174/138920208786241199

**Published:** 2008-11

**Authors:** Xinmin Li, Richard J Quigg, Jian Zhou, Weikuan Gu, P Nagesh Rao, Elaine F Reed

**Affiliations:** 1 Clinical Microarray Core, Department of Pathology & Laboratory Medicine, University of California at Los Angeles, 1000 Veteran Ave., Los Angeles, CA 90095, USA; 2 Department of Medicine, Biological Sciences Division, the University of Chicago, 5841 S. Maryland Ave., Chicago, IL 60637, USA; 3 Department of Orthopedics Surgery, Campbell Clinic, University of Tennessee Health Science Center, Memphis, TN 38163, USA

**Keywords:** Microarray, AmpliChip CYP450, MammaPrint, tissue of tumor origin, AmpliChip p53, navigenics, 23andMe, deCODE genetics.

## Abstract

Microarray-based clinical tests have become powerful tools in the diagnosis and treatment of diseases. In contrast to traditional DNA-based tests that largely focus on single genes associated with rare conditions, microarray-based tests are ideal for the study of diseases with underlying complex genetic causes. Several microarray based tests have been translated into clinical practice such as MammaPrint and AmpliChip CYP450. Additional cancer-related microarray-based tests are either in the process of FDA review or under active development, including Tissue of Tumor Origin and AmpliChip p53. All diagnostic microarray testing is ordered by physicians and tested by a Clinical Laboratories Improvement Amendment-certified (CLIA) reference laboratory. Recently, companies offering consumer based microarray testing have emerged. Individuals can order tests online and service providers deliver the results directly to the clients *via *a password-protected secure website. Navigenics, 23andMe and deCODE Genetics represent pioneering companies in this field. Although the progress of these microarray-based tests is extremely encouraging with the potential to revolutionize the recognition and treatment of common diseases, these tests are still in their infancy and face technical, clinical and marketing challenges. In this article, we review microarray-based tests which are currently approved or under review by the FDA, as well as the consumer-based testing. We also provide a summary of the challenges and strategic solutions in the development and clinical use of the microarray-based tests. Finally, we present a brief outlook for the future of microarray-based clinical applications.

## INTRODUCTION

Microarray-based clinical tests enabling the simultaneous detection of multiple genotypes and disease markers are expected to become a significant part of clinical diagnostic testing in the future, and hold promise in improving disease diagnosis, risk stratification, and selection and optimization of drug regimens [1]. Microarray-based tests are superior to traditional DNA-based tests or histopathologic assays because of their unique ability to simultaneously measure the relative expression level of a large number of clinically relevant genes, or to genotype a large number of allelic variants at one or more loci at once. These features are essential for the accurate diagnosis and management of diseases with underlying complex genetic causes. Recently, the “Critical Path to New Medical Products” by the U.S. Food and Drug Administration (FDA Whitepaper, 2004) and the Draft Guidance on *In Vitro* Diagnostic Multivariate Index Assays (IVDMIA) (Draft Guidance for Industry, Clinical Laboratories, and FDA Staff, 2006) have identified genomic technologies as a crucial component in advancing medical product development and personalized medicine [2].

Although microarray technology has led to many research discoveries which have laid the ground work for evaluating disease susceptibility, diagnoses, and prognoses [3-5], the translation from research to clinical utility has been slower than the microarray community anticipated, largely due to technical, clinical and marketing challenges. Several driving forces, including the need to diagnose common complex genetic diseases and the increased application of microarray technology to drug development, drug safety and efficacy have pushed microarray technology to the forefront of clinical practice. The AmpliChip CYP450 from Roche and MammaPrint from Agendia are the first FDA approved microarray-based tests for diagnostic applications. It is predicted that application of these tests to disease diagnosis, risk stratification and therapeutics will trigger an exponential increase of other microarray-based tests in the coming years.

## CURRENT STATUS OF MICROARRAY-BASED TESTING

### 1 Clinical Microarray Testing

#### AmpliChip CYP450 Test

**Fig. (1):**
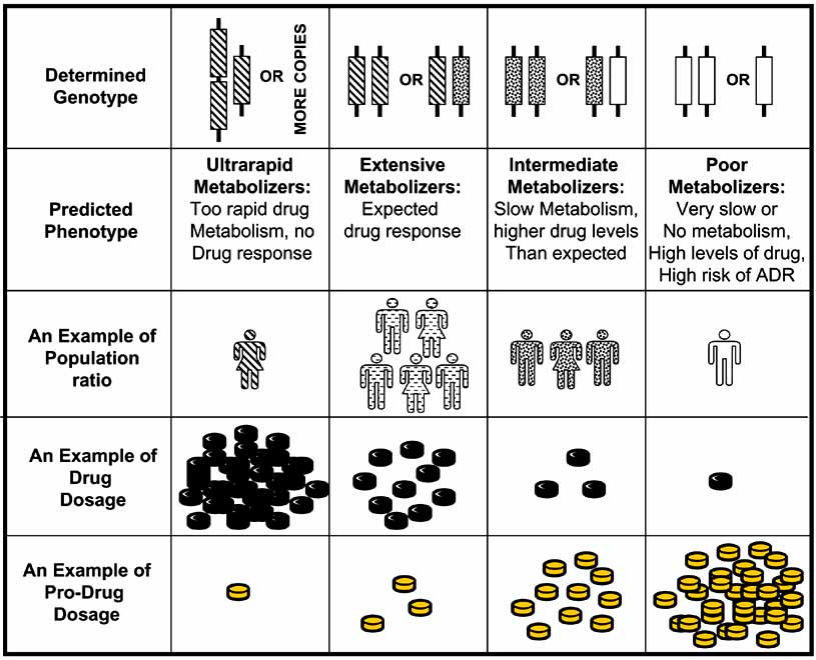
Schematic presentation of AmpliChip CYP450 test: from determined genotype, predicted phenotype to optimized drug dose.

Drug effectiveness and toxicity vary substantially across individuals. There is increasing evidence regarding the importance of genetic variation in influencing drug metabolism and response to therapy. DNA sequence variations in genes for drug metabolizing enzymes, like the Cytochrome P450 family, have major effects on the efficacy or toxicity of a drug [6]. The AmpliChip CYP450 test is the first FDA-approved, genotyping-based diagnostic test, which analyzes patient genotypes for two highly polymorphic cytochrome P450 (CYP) genes, CYP2D6 and CYP2C19. These genes regulate the metabolism of approximately 25 percent of all prescription drugs as summarized in Table **1** [7,8]. The test genotypes 29 polymorphisms and mutations in the CYP2D6 gene and two polymorphisms in the CYP2C19 gene. Based on a patient's CYP2D6 and CYP2C19 genotype, there are four predicted phenotypes: poor, intermediate, extensive or ultrarapid metabolizers. This information provides physicians with added knowledge when determining the appropriate drugs and doses, ultimately leading to improvement in patient outcome by reducing adverse drug reactions and improving drug efficacy (Fig. **1**). The AmpliChip CYP450 test has the potential to replace lengthy trial approaches and provide a quick and more efficient means for optimizing relevant drug dosage. However, to-date, there are no published studies to demonstrate improved patient outcome as a result of the AmpliChip test. 

As of June 1, 2007, eight laboratories offer the test in the United States (http://www.amplichip.us/physicians/prescribingthetest.php). Physicians prescribe the AmpliChip^®^ CYP450 Test and send 3-5 ml of patient’s blood sample collected in an EDTA anticoagulant (lavender cap) tube to one of the laboratories. The results are received within several days after receipt of the blood sample by the lab. The test costs between US$600 and US$1300 and is currently not covered by third-party insurers. However, the test is done only once in a person’s lifetime and provides a permanent genotype record which can guide physicians in effectively prescribing appropriate dosages of relevant medications in genetically susceptible patients.

#### MammaPrint^®^ Breast Cancer Test

**Fig. (2):**
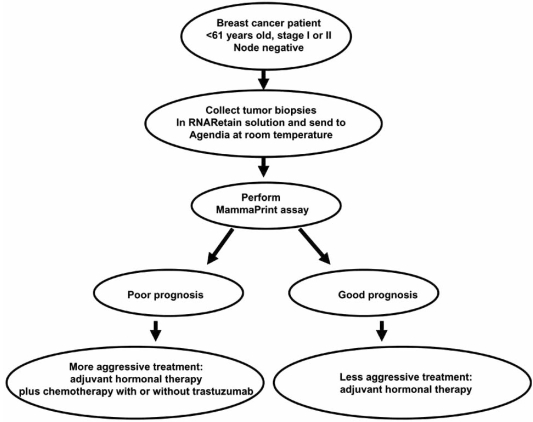
The outline of the MammaPrint test and subsequent treatment. Adjuvant hormonal therapy should be given to patients with estrogen-receptor-positive disease.

Sixty-five percent of women diagnosed with invasive breast cancer have lymph node-negative disease at diagnosis [9]. Of these women, 85% are expected to survive and be free from distant metastasis for 10 years [10]. However, most patients are treated with relatively nonspecific cytotoxic chemotherapy or hormonal therapy because physicians are unable to identify patients who will have the greatest risk of recurrence and most likely to benefit from the therapy [11]. Thus, a major goal is to stratify patients for chemotherapy or hormonal therapy based on the patients’ risk of recurrence. MammaPrint is the first FDA approved, gene expression-based prognostic test which assess patients’ risk for distant metastasis in women under age 61 with Stage I-II lymph node negative breast cancer. The test was developed by Agendia, a gene expression-based diagnostic company in Amsterdam (http://www.agendia.com/), and was approved by the FDA in February 2007 as an Agendia-only offered service. The MammaPrint test measures the expression levels of 70 genes in a sample of a woman's surgically removed breast tumor, whose activity confers information about the likelihood of tumor recurrence. A specific algorithm is then used to produce a score that determines whether the patient is deemed low risk or high risk for cancer metastasis [12,13]. The high-risk patients may then be stratified for more aggressive therapy (Fig. **2**). Agendia has recently shown that the MammaPrint test is also useful for postmenopausal breast cancer patients, and thus is seeking additional FDA clearance to include patients over the age of 60.

The MammaPrint test has been validated in an independent dataset, and shown to be superior to currently available clinicopathologic prognostic indicators [14]. The benefit of MammaPrint in therapeutic decision-making is undergoing evaluation in the MINDACT trial (Microarray in Node negative Disease may Avoid Chemotherapy), which is currently enrolling patients in Europe (www.cancer.gov/clinical trials) [15]. The test is not available in the United States. Users can order MammaPrint Specimen Collection and Transportation Kits online (http://usa.agendia.com/en/ordering.html), by placing the tumor biopsy specimens in RNARetain, an RNA stabilizing solution, which was also FDA-approved for this function in June 2007, and then send to Agendia by FedEx. Results are delivered electronically within 10 days at a cost of $3,200. 

#### Pathwork™ Tissue of Origin Test

**Fig. (3):**
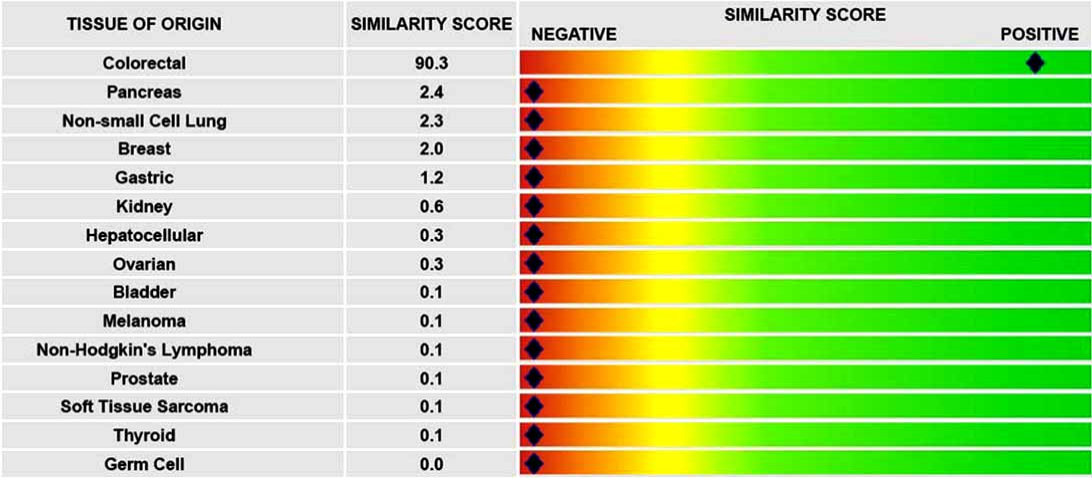
An example of Pathwork tissue of origin test report. The test produced a similarity score of >30 for colorectal tissue and <5 for other tissues, indicating that the tumor was originated from colorectal. Fifteen tested tissues are listed on the left.

Making the correct diagnosis of the origin of a tumor is critical for selection of the appropriate treatment strategy of any cancer [16]. Using standard pathological techniques, it is estimated that up to 5 to 10% of all tumors may actually be misclassified [17,18], and nearly 2 to 5% of all tumors need to be diagnosed for their origin [19]. The Tissue of Origin test is a gene-expression based diagnostic assay for determining the tissue of tumor origin for poorly differentiated or undifferentiated tumor specimens. Based on expression levels of 1550 selected and 110 control genes, the test compares the similarity of tumors of unknown origin to cancers from one of 15 tissues of known origin by using proprietary normalization and classification algorithms. A similarity score is then assigned to each of 15 tumor types in a physician-friendly report. A Similarity Score > 30 is considered “positive,” and a score <5 is called “negative.” Values between 5 and 30 are designated as “no call” for that tissue (Fig. **3**). This test is expected to aid in the diagnosis of the tissue of origin and help physicians select appropriate tissue-directed therapies.

The performance of the test has been validated using 477 clinical samples in a multi-center clinical trial [20]. Four separate laboratories have recently tested the reproducibility of Tissue of Origin gene-expression signatures on 60 archived tissue specimens from poorly and undifferentiated tumors. Cross-laboratory comparisons showed correlation coefficients of 0.95 to 0.97 among measurements [21]. Tissue of Origin is currently under FDA review. On April 22, 2008, Pathwork Diagnostics announced the launch of the Pathwork Tissue of Origin Test through its CLIA-certified Laboratory using Affymetrix-based PathChip™.

#### AmpliChip p53 Test

The tumor suppressor gene *p53* is one of the most frequently mutated genes in human cancer (http://www-p53.iarc.fr/index.html). Emerging research reveals that p53 mutations are associated with the prognosis of cancer patients and can predict response to therapy in many cancers [22]. Thus, the identification of *p53* somatic mutations will be useful for predicting patient outcome and response to treatment. The AmpliChip p53 test is a microarray-based resequencing test currently under development by Roche Molecular Diagnostics. The test is designed to detect single nucleotide substitutions and 1 bp deletions in the entire coding region and the flanking splice sites of exons 2-11 of the *p53* gene in either formalin-fixed paraffin-embedded tissue (FFPE) or freshly frozen tissue [23]. The AmpliChip p53 test queries for the presence of sequence alterations through comparative analysis of the hybridization pattern of a series of probes to sample DNA and wild-type reference DNA. Compared to conventional DNA sequencing, the highly redundant probe tiling approach is able to detect a significantly lower abundance of *p53* mutations in samples which contain mixtures of normal and tumor tissue without the need for microdissection.

Several clinical research studies using the AmpliChip p53 test are underway. One such study analyzed over 700 breast cancer FFPE samples, of which *p53* mutations were found in ~40% of patients. From this cohort, 272 samples were also analyzed by single strand conformation polymorphism (SSCP)-sequencing. These results revealed a 94.1% concordance between the AmpliChip p53 test and SSCP-sequencing. Of the 16 discordant samples (6%), it was found that these samples contained insertion mutations or had > 2-bp deletions which the AmpliChip p53 is not designed to detect [24]. Another ongoing study is the investigation of the predictive value of the AmpliChip p53 test in a clinical trial of patients on capecitabine and docetaxel with or without trastuzumab in locally advanced breast cancer. As an initial step, Roche is focusing on the use of AmpliChip p53 as a prognostic and therapy selection test in breast cancer. 

#### Chromosomal Abnormality Test

Many clinically delineated syndromes are caused by chromosomal abnormalities, such as submicroscopic deletions or duplications [25]. Fluorescence *in situ* hybridization (FISH) with chromosome-specific probes has been an effective tool for the diagnosis of chromosomal abnormalities. However, due to the limited coverage of FISH probes and their detection sensitivity, many pathogenic chromosomal aberrations are still undetected. The array-based comparative genomic hybridization (aCGH) has proven to be an invaluable tool to assess chromosomal aberrations at a higher resolution and more comprehensive coverage [26]. In fact, it has recently become the most commonly used, microarray-based test in a clinical setting. The tests broadly fall into two categories: BAC-based aCGH and oligo-based aCGH. Among these tests, some interrogate specific regions associated with known chromosomal abnormalities while others detect known and hitherto unknown aberrations at large.

Several companies offer aCGH services in diagnostic applications, including Combimatrix Molecular Diagnostics, Signature Genomic Laboratories and Gene DX. UCLA Clinical Microarray Core recently joins the team by providing genomic analysis on Affymetrix Genome-Wide Human SNP Array 6.0 platform. Driven by the clinical demands, new generations of aCGH platforms are rapidly evolving. On April 8, 2008, Combimatrix Molecular Diagnostics launched its new version of high-density BAC array test (BAC HD Scan), comprised of 2437 unique large-insert clones designed to both interrogate specific regions associated with more than 125 known genetic disorders and enable screening for unknown abnormalities with an average size of 1 Mb (median 432 Kb) resolution across the entire genome. To compete for better resolution, Signature Genomic Laboratory has moved from their traditional BAC aCGH to Oligo aCGH. Recently released SignatureChipOS includes 105,000 oligonucleotides covering every region known to be involved in cytogenetic abnormalities (over 150 syndromes) with a maximum probe spacing of one probe every 35 kb throughout the genome and one probe every 10 kb in clinical regions. Both BAC HD Scan and SignatureChipOS emphasize identifying clinically known abnormalities. Affymetrix has recently released the Genome-Wide Human SNP Array 6.0, which includes more than 1.8 million markers on a single array with an inter-marker distance of 696 base pairs, providing unprecedented power to detect even very small gains and losses globally. This unbiased high-density oligo array will allow for sensitively detecting all known abnormalities with defined loci of interest as well as those that have not been clinically established. As studies using such high-density oligo arrays yield a dataset that can differentiate benign genomic variants from those associated with a disease state, the global approach of high-density oligo aCGH will become a dominant player in this rapidly expanding diagnostics market.

### 2 Consumer-Based Microarray Testing

#### Navigenics Health Compass

Confidentiality is a substantial concern for genetic tests that can become part of an individual’s personal health information. There is concern that health insurance and even job opportunities could be jeopardized if evidence of genetic risk factors for disease were to become a part of a patient’s medical record. To address this issue, web-based genetic testing services are emerging. One such test is Navigenics Health Compass developed by Navigenics Inc (http://www.navigenics.com/). The Health Compass is a web-based prognostic test to predict risk for 18 prevalent genetic diseases including diabetes, obesity, cancer and heart disease (Table **2**). Using the combined data from over 4000 scientific papers describing associations between single nucleotide polymorphisms (SNPs) and certain diseases, Navigenics identified a subset of SNP markers for each of the 18 diseases. Based on the presence or absence of specific SNPs, Navigenics developed an algorithm to estimate the risk of a healthy person developing a particular disease. Consumers can have the test performed without initial involvement of a physician intermediary by ordering the test kit online, and then sending a sample of their saliva to Affymetrix Inc. for high-density SNP genotyping. Navigenics staff then perform in-depth analysis using the proprietary SNP-disease association information and post the results on a secure internet site, which the customers can login and access the data. Following the test, a licensed genetic counselor from Navigenics will provide free consultation by phone to interpret the test results. Navigenics’ medical partners will provide advice on strategies to possibly reduce the risk of developing the disease. During the entire process, customers have full control of their medical information and can decide if and to whom to disclose the information. The test was officially launched on April 8th, 2008 at a cost of $2500.

#### 23andMe

A second internet-based microarray test was launched by 23andMe on November 19, 2007. The test combines Illumina genotyping with a set of tools developed by 23andMe to depict each customer's personal data within the context of the latest scientific findings, environmental and other factors that contribute to variation in human traits and conditions. The test reports risk for 16 diseases and physical traits (Table **2**). In addition, customers can use the web-based tools to trace ancestry and compare DNA similarity with family members and friends, or compare other published genetic information with their own genotype. In contrast to Navigenics, 23andMe puts less weight on clinical prognosis, but instead provides personalized genetic information. The sample collection and handling procedures are similar to those required by Navigenics. The analysis is completed in two to four weeks and at which time customers will be able to use their private login to access their genome data, learn their risks for certain diseases, explore their ancestry, and compare themselves to friends and family members. The test is now available for $999.

#### deCODE Genetics

Officially launched on November 16, 2007 by deCODE Genetics in Iceland, a pioneer in disease gene hunting, deCODEme is the first internet-based microarray test [27]. The overall concept of deCODEme is same as 23andMe, but uses buccal cells obtained through cheek swabbing rather than saliva. The deCODEme test calculates genetic risk for 18 complex genetic diseases based on the current literature, reconstructs the geographical distribution of the customer’s ancestors and compares DNA similarity with selected family and friends. The test also adopts Illumina technology to survey over one million SNPs. The key features and differences of these 3 internet-based microarray tests are summarized in Table **2**.

## CHALLENGES AND STRATEGIC SOLUTIONS

Despite the numerous research discoveries and tremendous interest in using the microarray-based tests for disease diagnosis and patient management, few have been translated into clinical practice. This inaction is attributable to technical, regulatory and marketing challenges. Table **3** summarizes some of the major challenges and strategic solutions for the development and clinical use of the microarray-based tests. Here, we discuss several key marketing-related challenges.

One major challenge for marketing some of those microarray-based tests is the lack of a solid scientific foundation. For example, the above discussed three customer-based testing interrogate only about one million SNPs (or less), representing the common SNPs occurring in at least 5% of the population, whereas rare SNPs that occur at a frequency of 1% are underrepresented. Given the fact that there are an estimated 15 million SNPs along the 3 billion bases of the human genome [28], the current tests are incomplete and biased toward detection of common variations. Furthermore, most of the disease-associated SNP markers identified so far exert relatively small effects and involve interactions with other genetic factors and environmental conditions which are still poorly defined. In addition, it is estimated that 20% of differences in gene activity are attributed to copy-number variants that are not covered by SNP variations. All these factors represent significant limitations and raise the question as to whether these tests are sufficiently comprehensive to be accurate or worthwhile. Furthermore, even if the test provides some useful information, are patients going to change their lifestyle in order to delay or prevent the disease? If patients do modify their lifestyle, will that lead to a decreased risk of disease? The paucity of prevention studies showing the benefit of genetic testing represent a significant barrier to translating genetic testing to clinical practice. In an effort to address these challenges, on January 22, 2008 ,an international research consortium unveiled “The 1,000 Genomes Project” which will involve sequencing the genomes of at least one thousand people from around the world to create the most detailed and medically useful picture of human genetic variation [29]. By sequencing these 1,000 genomes, rare SNPs occurring at a 1% frequency as well as copy number variations will be uncovered. This ambitious effort will significantly alleviate the current problems associated with incomplete and biased SNP arrays and will provide a comprehensive picture of genetic variations. Ultimately this should lead to a better understanding of how people are predisposed to or protected from disease. With regards to the beneficial evidence of such tests, more coordinated, large-scale clinical trails are needed. Agendia is aggressively moving toward this direction by organizing the MINDACT trial to evaluate the benefit of MammaPrint in therapeutic decision-making.

Other marketing challenges include cost and confidentiality. These tests currently cost between $600 and $3500. Health insurers are unlikely to cover the costs of these tests until studies prove their value and can link them to improved health. Given that the aforementioned scientific challenges are being addressed, it is likely that these tests will be covered by health insurance companies in the coming years. A patient’s right to privacy is another concern. Many people worry that the results of such tests would reveal a genetic risk of disease and disclosure of this information may have a negative impact on their health insurance and employment. In this regard, local governments have taken protective actions with 34 states and now having non-discrimination legislation covering genetic information. A similar federal law has already been passed by the House, and awaits approval in the Senate. 

One remaining hurdle when adopting such tests is community awareness. At this point, the population at-large is unfamiliar with such tests and few physicians are familiar in the intricacy of test interpretation and how to advise patients based on the results. Consequently, health care providers are reluctant to prescribe microarray-based tests given that they do not fully appreciate their value at this point. An urgent task ahead is to develop supporting information systems which offer educational and consultation programs for physicians and other health care providers as well as the general population to facilitate the adoption and use. Ultimately, these educational programs will display the impact of microarray-based tests in therapeutic decision making. 

## FUTURE PERSPECTIVE

After a long journey, the year 2007 saw significant advancements in microarray-based clinical testing. Several microarray-based tests have now come to fruition and entered the clinical lab, the ultimate home of microarray technology (Table **4**). Many more are under development. TessArae LLC is actively performing a TessArray testing which will simultaneously detect and identify hundreds of strains of natural and emergent viral and bacterial pathogens; bioMérieux is working on HIV genotyping and microbial contamination testing; Skyline Diagnostics is developing a test for acute myeloid leukemia testing; a cancer DSA pipeline is being established by ALMA. On March 12, 2008, Mobidiag, a Finland-based biotech company, launched its first microarray-based test for simultaneous identification of eight different human herpesviruses. Guided by these early clinical practices and driven by the explosion of new discoveries and marketing demands, the next wave of microarray-based tests will soon be upon us. There still is, however, considerable work ahead before these tests can impact clinical practice to the magnitude their potentials would allow.

## Figures and Tables

**Table 1:** Clinically Relevant Drugs Metabolized by CYP2D6 and CYP2C19

CYP2D6			
Beta Blockers	Antidepressants	Antipsychotics	Others
Carvedilol Metoprolol Propafenone Timolo	Amitriptyline Clomipramine Desipramine Imipramine Paroxetine Venlafaxine	Haloperidol Risperidone Thiroridazine	Atomoxetine Codeine Dextromethorphan Flecainide Mexiletine Ondansetron Tamoxifen Tramadol
**CYP2C19**			
**Proton Pump Inhibitors **	**Antidepressants**	**Anti-epileptics**	**Others**
Omeprazole Lansoprazole Pantoprazole	Amitriptyline Clomipramine	Diazepam Phenytoin Phenobarbitone	Cyclophosphamide Progesterone

**Table 2:** Comparison of Three Web-Based Microarray Tests

Company Name	Navigenics	23andMe	deCODE Genetics
Test Name	Health Compass		deCODEme
Genotyping platform	Affymetrix	Illumina	Illumina
# of SNPs interrogated	One million	0.58 million plus some proprietary SNPs	One million
Date of service launched	April 8, 2008	Nov. 19, 2007	Nov. 16, 2007
Sample type	Saliva	Saliva	Buccal swab
# of predicted diseases & physical traits	18(Alzheimer's disease, Breast cancer, Celiac disease, Colon cancer, Crohn's disease, Type 2 diabetes, Glaucoma, Graves' disease, Heart attack, Lupus, Macular degeneration, Multiple sclerosis, Obesity, Osteoarthritis, Prostate cancer, Psoriasis, Restless legs syndrome, Rheumatoid arthritis)	16 (Breast Cancer, Crohn's Disease, Heart Attack, Multiple Sclerosis, Obesity, Prostate Cancer, Restless Legs Syndrome, Rheumatoid Arthritis, Type 1 Diabetes, Type 2 Diabetes, Venous Thromboembolism, Alcohol Flush Reaction, Bitter Taste Perception, Earwax Type, Lactose Intolerance, and Muscle Performance)	20 (Age-related Macular Degeneration, Asthma, Alzheimer's Disease, Atrial Fibrillation, Breast Cancer, Celiac Disease, Colorectal Cancer, Exfoliation Glaucoma XFG, Crohn's Disease, Multiple Sclerosis, Myocardial Infarction, Obesity, Prostate Cancer, Psoriasis, Restless Legs, Rheumatoid Arthritis, Type 1 Diabetes, Type 2 Diabetes, eye color and hair color)
Follow-up strategy	Offer free genetic consultation	Provide referrals to genetic counselors	Provide referrals to genetic counselors
Aim of the test	Emphasis on disease prognosis	Emphasis on providing personalized information	Emphasis on providing personalized information
Cost	$2500	$999	$985

**Table 3:** Current Challenges and Strategic Solutions for the Development and Clinical Use of Microarray-Based Tests

Technical Challenges for the Development of Microarray-Based Tests	
Technical Challenges	Strategic Solutions
Robust analytical performance across laboratories with different instrument, different lots of reagents and different operators.	Develop assay-specific, tissue-specific and array platform –specific data normalization algorism, including identifying a suitable panel of internal control genes for normalization, to ensure the difference in the gene expression is mostly due to difference in the biology of the tissue samples.
Reproducible measurements for the same subject under the same conditions	Standardize all procedures and automate operations to minimize human intervention
Appropriate quality control materials specific to microarray platforms	Develop standardized quality control kit for different microarray platforms
Random measurement bias associated with sample collection and DNA/RNA quality	Specify detailed, internationally uniformed sample collection procedures and requirements for each test; to quantify and standardize the requirements for DNA/RNA quality (both quantity and integrity)
Systematic measurement bias associated with study design, population and geographical location	Obtain samples representative of the population for whom the test is intended. Offer panels that provide high detection rates for all ethnic groups
Identifying the appropriate set of genes that maximize the detection power to distinguish the outcome classes of a disease	Develop new algorism and software to effectively separate and balance biological information and noise. Use representative and large sample population
**Marketing Challenges for the Clinical Use of Microarray-Based Tests**	
**Marketing Challenges**	**Strategic Solutions**
Scientific foundation of the tests are based on current literature, which is incomplete and biased	The 1000 Genome Project announced by the International Consortium on January 22, 2008 will effectively address this challenge
Absence of a sufficient scientific evidence of benefit for a clinical use	Perform clinical trails to document the benefit of the use of tests
High cost and uncertainty about insurance coverage	Address the issue of cost by technological renovation and advancement.
Privacy issue	Promote the passage of anti-discriminatory legislation and offer web-based testing in which only patients can access and decide whether and how to distribute the information
Difficulty to obtaining FDA approval for microarray-based tests	FDA has fully realized the importance of such tests. The approval will be accelerated after establishing appropriate regulatory rules
Lack of Healthcare providers and community educational programs	Implement Clinician education at all levels and invest in direct to consumer advertising

**Table 4:** A Summary of Currently Available or Upcoming Microarray-based Tests

Testing	Specimens	RNA/DNA	Measured	Number of Genes/SNPs	Availability	Status of FDA-Approval
**Clinical Testing**						
AmpliChip CYP450	Blood	DNA	CYP450 SNPs	31 + controls	Yes	Yes
MammaPrint	Tumor	RNA	Relevant genes	70	Yes	Yes
Tissue of Origin	Tumor	RNA	Relevant genes	1550 + 110 controls	No	Under review
AmpliChip p53	Tumor	DNA	p53 mutations	coding region + splice sites	No	No
aCGH	Blood/Tissues	DNA	Chromosomal abnormalities	Up to 1.8 million markers	Yes	No
**Consumer-Based Testing**						
Health Compass	Saliva	DNA	SNPs	1 million	Yes	No
23andme	Saliva	DNA	SNPs	0.58 million + proprietary	Yes	No
deCODEme	Buccal swab	DNA	SNPs	1 million	Yes	No
